# Aversion to Desflurane and Isoflurane in Sprague-Dawley Rats (*Rattus norvegicus*)

**DOI:** 10.3390/ani10060950

**Published:** 2020-05-30

**Authors:** Katrina Frost, Maaria Shah, Vivian S.Y. Leung, Daniel S.J. Pang

**Affiliations:** 1 Faculty of Veterinary Medicine, University of Calgary, Calgary, AB T2N 4Z6, Canada; kfrost@ucalgary.ca; 2 Faculty of Science, University of Calgary, Calgary, AB T2N 1N4, Canada; maaria.shah@ucalgary.ca; 3 Faculty of Veterinary Medicine, Université de Montréal, Saint-Hyacinthe, QC J2S 2M2, Canada; vleung01@alumni.uoguelph.ca

**Keywords:** euthanasia, rats, animal welfare, refinement, aversion, isoflurane, desflurane, aversion-avoidance

## Abstract

**Simple Summary:**

Euthanasia is one of the most commonly performed procedures in laboratory rodents, as the majority of animals are killed upon project completion or when humane endpoints have been reached. Overdose with carbon dioxide gas remains a widely used killing method, despite evidence it is aversive to rodents. The inhalant anesthetic isoflurane is a refinement to overdose with carbon dioxide, but also elicits aversion in rodents. The inhalant anesthetic desflurane has a faster onset of action than isoflurane and may therefore offer further refinement. In this study, rat aversion to desflurane and isoflurane was compared. Isoflurane and desflurane were similarly aversive; however, desflurane exposure resulted in a shorter time to achieve recumbency, shortening any period of potential distress. Therefore, desflurane represents a refinement over the use of isoflurane.

**Abstract:**

Carbon dioxide and isoflurane are widely used for killing rats, yet may not truly achieve “euthanasia”, because they elicit aversion. The inhalant anesthetic desflurane is faster acting than isoflurane, representing a potential refinement. Using an aversion-avoidance paradigm, 24 rats were exposed to isoflurane or desflurane (*n* = 12 per group) at initial exposure. Fourteen rats were then re-exposed to isoflurane or desflurane (*n* = 7 per group), after a 7 days washout period. Initial exposure: time to recumbency was faster for desflurane than isoflurane (*p* = 0.0008, 95% CI [-12.9 to 32.6 s]), with 9/12 and 6/12 rats becoming recumbent, respectively. At initial exposure, there was no difference between groups in time to withdrawal (*p* = 0.714). At re-exposure, all rats withdrew and no rats became recumbent. Time to withdrawal at re-exposure did not differ between treatment groups (*p* = 0.083). Compared to initial exposure, time to withdrawal during re-exposure was similar for isoflurane (*p* = 0.228) and faster with desflurane (*p* = 0.012, 95% CI [19.1 to 49.5 s]). Isoflurane and desflurane are similarly aversive, with aversion increasing at re-exposure. The shorter time from exposure to recumbency with desflurane indicates that any distress is of a shorter duration when compared with isoflurane.

## 1. Introduction

Euthanasia is one of the most commonly performed procedures in laboratory rodents, as the majority of animals are killed upon project completion or when humane endpoints have been reached. Overdose with carbon dioxide gas (CO_2_) remains a widely used killing method, despite evidence it is aversive to rodents [1,2,3]. It remains popular because it is economical, easy to use, quick acting, relatively safe to personnel and can be applied to multiple animals at the same time. The American Veterinary Medical Association and Canadian Council on Animal Care have classified CO_2_ as an acceptable killing method, provided that certain conditions are met (e.g., adherence to recommended technique, properly functioning equipment, scientific justification) [4,5]. A refinement to CO_2_ is the use of an inhaled volatile anesthetic, such as isoflurane [6,7]. However, it appears that the volatile anesthetics that have been studied to date (isoflurane, sevoflurane, desflurane) also induce a degree of aversion and this can increase upon re-exposure [6,7,8,9].

The volatile anesthetic desflurane has unique properties that make it an appealing alternative to both isoflurane and sevoflurane. It leads to a more rapid loss of consciousness, as reflected by a blood-gas solubility coefficient approximately 30–60% lower than that of sevoflurane and isoflurane, respectively. This is counterbalanced by a pungent smell and airway irritation that is less well tolerated than sevoflurane and isoflurane in humans [10]. Previous work in rats (Wistar) and mice (BALB/c) exposed to a test chamber pre-filled with desflurane showed it to be aversive [8].

The aim of this study was to compare desflurane and isoflurane using a gradual fill aversion-avoidance testing method, during both initial and re-exposure to each anesthetic. Aversion-avoidance testing has been successfully used in rats to identify and quantify aversion to isoflurane, sevoflurane and CO_2_ [6,7]. The primary objective was to compare the number of animals becoming recumbent with each anesthetic and the time to achieve recumbency. A secondary objective was to compare the time to withdraw from each anesthetic agent. We hypothesized that desflurane would result in more animals becoming recumbent and a shorter time to recumbency.

## 2. Materials and Methods

The Health Sciences Animal Care Committee of the University of Calgary, operating under the auspices of the Canadian Council on Animal Care, approved the experimental protocol (AC11-0044).

### 2.1. Subjects and Housing

Sprague-Dawley rats, 8–15 weeks old, 14 male (median 288 (range 262–375) g) and 14 female (247 (222–259) g), were purchased from Charles River Laboratories (Montreal, QC, Canada), or obtained as surplus stock from the University of Calgary (one generation from animals originally purchased from Charles River Laboratories and not previously used in any experiments). Purchased animals were acclimatized to the housing facility for at least one week. Animals were housed in pairs or triplets in a conventional housing system in a climate-controlled room (23–24 °C humidity 20%, 139–279 Lux, 12 h light-dark cycle (lights on: 0700–1900)). Each polycarbonate cage (47 × 25 × 21 cm; RC88D-UD, Alternate Design Mfg and Supply, Siloam Springs, Arizona, USA) included bedding (Aspen Chip, Nepco, Warrensburg, NY, USA), sizzle paper (Spring Fill Industries, Northbrook, IL, USA), a nest box or a tube (Bio-Serv, Flemington, NJ, USA), free access to food (Prolab RMH 2500, Labdiet, St. Louis, MO, USA) and tap water ad libitum. Health checks were performed at least twice daily throughout the experimental period. Sentinel rats were in use: negative for rat parvoviruses Toolan H1 virus, Kilham rat virus, rat minute virus and protoparvovirus NS-1, rat sialodacryoadenitis virus, rat theilovirus, *Pneumocystis carnii*, Sendai virus, pneumonia virus of mice, reovirus, *Mycoplasma pulmonis*, Lymphocytic choriomeningitis virus, adenovirus, hantavirus, *Encephalitozoon cuniculi*, cilia-associated respiratory bacillus, rat rotavirus, *Bordetella bronchiseptica*, *Corynebacterium kutscheri*, *Klebsiella oxytoca*, *Klebsiella pneumoniae*, *Rodentibacter pneumotropicus*, *Pseudomonas aeruginosa*, *Staphylococcus aureus*, *Streptococcus* β hemolytic and *Streptococcus pneumoniae*, *Proteus mirabilis*, *Salmonella* and other bacteria, and endo- and ectoparasites.

### 2.2. Experimental Apparatus

**Figure 1:**
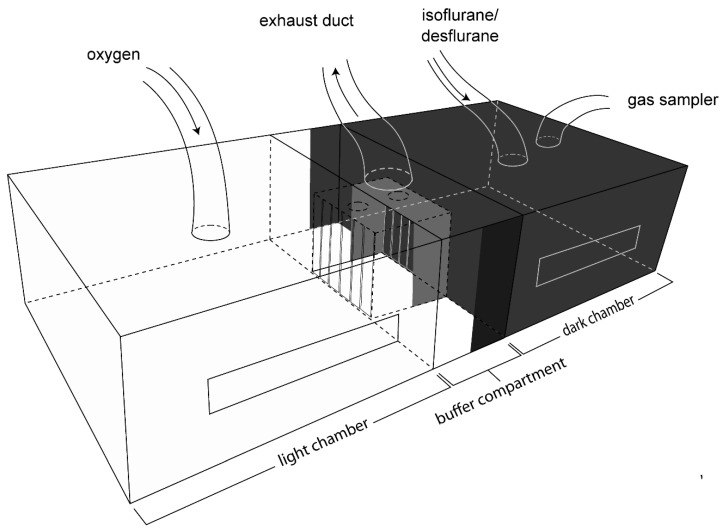
Illustration of the aversion-avoidance testing apparatus.

The Plexiglas light-dark aversion-avoidance box (Figure 1), provided by D. Weary [6], was comprised of two chambers of identical size (28 × 31 × 14 cm each), which were connected by a smaller middle buffer compartment (11 × 31 × 14 cm). Exhaust from the buffer compartment was actively scavenged. The chambers were wrapped with white (light chamber) or black (dark chamber) opaque plastic. Observation slits (3 × 17 cm) were cut into the opaque plastic on one side of each chamber. The buffer compartment had a doorway (10 cm × 9 cm) on each side leading into each chamber. To prevent diffusion of gas between the chambers, the doorways were covered with overlapping strips of flexible plastic. Those of the dark chamber were black opaque strips to minimize light contamination (light intensity in the dark chamber did not exceed 2.5 Lux). Maintenance of isolated environments (anesthetic gas vs. room air) between the dark and light chambers was confirmed with a gas analyzer (sample rate 50 mL/min, LifeWindow LW6000V, Digicare Biomedical Technology Inc., West Palm Beach, FL, USA).

The Plexiglas lid covering the entire box had 4 holes: two centered above the dark chamber, one for the gas sampling line (1.3 cm diameter) and one for gas delivery (1.5 cm diameter), a hole (4.5 cm diameter) centered above the buffer chamber to scavenge gas, and a hole (1.5 cm diameter) centered above the light chamber for gas delivery. Each chamber was connected to its own oxygen tank, with flow meters calibrated with a respirometer (Wright Mark 8, Nspire, Longmont, CO, USA).

The light chamber was illuminated by centering a dimmable desktop LED lamp (Astra LED Desk lamp, Workrite, Petaluma, CA, USA) 58 cm above the chamber, with a light intensity range of 279–343 lux (measured at each corner of the light chamber using a light meter (Autometer VF, Konica Minolta, Osaka, Japan)), comparable to light levels in the housing room.

### 2.3. Habituation and Re-Habituation

Rats were habituated to handling (10 min, twice daily) and to the light/dark box. Habituation to the box lasted between four to seven days. During habituation, the LED lamp and gas sampler were switched on and oxygen was administered into both chambers, starting at 1 L/min on day one, 2 L/min on day two, 3 L/min on day three and 4 L/min on days four to seven. On each day of habituation, five treats (Honey Nut Cheerios^TM^, General Mills, Inc., Golden Valley, Minnesota, USA) were placed in each chamber. Rats were always placed into the dark chamber first and allowed to explore the entire box freely for 30 min. Bedding was placed in each chamber and was only changed when a different cage of rats was used. The box was cleaned (Windex Original, SC Johnson & Son, Brantford, ON, Canada), rinsed with tap water and dried between cages. Habituation was completed when: (1) rats crossed between the dark and light chambers at least five times and (2) rats consumed at least 9 out of the 10 treats within 30 min. Both requirements had to be met 3 times to prevent exclusion from the study.

Before the re-exposure experiment, rats were re-habituated to the light/dark box as described above, except that the criteria were considered satisfied the first time they were met (up to three days were allowed), and rats were excluded if they did not meet habituation requirements.

### 2.4. Initial Exposure and Re-Exposure

Rats were stratified by sex and block randomized (list randomizer, random.org) to one of two treatment groups: isoflurane or desflurane. The order of testing was not randomized: testing was performed according to the order in which rats met habituation criteria. All testing was performed between 1100 h–1800 h. On each testing day, the gas analyzer was tested for accuracy with a calibration gas (755571-HEL Calibration gas desflurane, CO2 agent; Datex Ohmeda, Louisville, KY, USA).

The procedure for initial and re-exposure testing was identical: five treats were placed in the light chamber, with oxygen delivered to both chambers at 4 L/min (33% of chamber volume per minute), immediately followed by placing rats individually in the dark chamber. Rats were able to explore and settle (defined as eating all treats and remaining in the dark compartment for at least 5 consecutive min). Up to 20 min was allowed for this to occur.

Once settled, the predetermined anesthetic was administered into the dark chamber. Desflurane (Tec 6 Plus, Datex Ohmeda, Louisville, KY, USA) and isoflurane were administered at 18% and 3.2%, respectively (2.2 × MAC of each agent) [11,12]. The maximum duration of the trial was 3 min. The trial ended at 3 min or when recumbency occurred, whichever was first. Rats were re-exposed to the same anesthetic seven days later. At the end of each experiment, rats were returned to their home cage, once ambulating normally and showing normal behaviors.

### 2.5. Data Collection

Trials were video-recorded (HC-W570 Hybrid O.I.S, Panasonic Full HD, UK and Ireland) through the observation slit in the dark chamber. During initial exposure of the first six rats to anesthetic (desflurane; *n* = 4, isoflurane; *n* = 2), an additional camera recorded the light chamber to capture possible escape behaviors (Table 1). This was out of a concern that should escape behaviors be observed, re-exposure could elicit significant distress. An observer (MS), blinded to treatment (anesthetic vaporizer covered and operated by a second observer (KF)), performed live observations (in case of video failure) and reviewed all video recordings to ensure timing accuracy. The following data were collected: total dwelling time, time to withdrawal, time to recumbency, and time from ataxia to recumbency (Table 2). The number of rats that became recumbent or withdrew was recorded.

### 2.6. Statistical Analysis

Most data did not approximate a normal distribution when assessed with the D’Agostino and Pearson test and non-parametric testing was applied throughout. The effect of treatment (isoflurane vs. desflurane) and exposure (initial vs. re-exposure) on time to withdrawal, time to recumbency, and time from ataxia to recumbency and total dwelling time were analyzed using a Mann–Whitney test. The effect of treatment and exposure on the number of rats becoming recumbent was analyzed using a Fisher’s exact test. Pooling data for analysis were considered where no effects over time (initial versus re-exposure) or between treatments were identified. Data were analyzed with commercial software (Prism 6 for Mac OS X, Version 6.0f, Graphpad Software, Inc. San Diego, CA, USA). A sample size of approximately 11 animals per treatment group was estimated for time to recumbency (alpha 0.05, 80% power, mean difference 20 s, SD 15 s) and proportion becoming recumbent (alpha 0.05, 80% power, proportion difference 0.5), respectively (G *Power 3.1.9.4, Germany) [6]. The number of rats used for re-exposure was reduced due to welfare concerns following the number of animals observed withdrawing from the dark chamber during initial exposure. *p*-values < 0.05 were considered significant. 95% confidence intervals (95% CI) for median differences are presented where available. Data are presented as median (range). Data supporting the results are available in an electronic repository: https://doi.org/10.7910/DVN/VIRZND.

## 3. Results

Before initial exposure testing, one animal from each treatment was excluded because they displayed ataxia in the light chamber (where the anesthetics were not introduced). Two animals (one from each treatment group) were excluded for not meeting habituation criteria. Therefore, the numbers of animals from which data were analyzed were: initial exposure; *n* = 12 (6 females, 6 males) per treatment group, re-exposure; *n* = 8 (4 females, 4 males) per treatment group. No escape behaviors were observed during initial exposure.

### 3.1. Time to Recumbency

**Figure 2:**
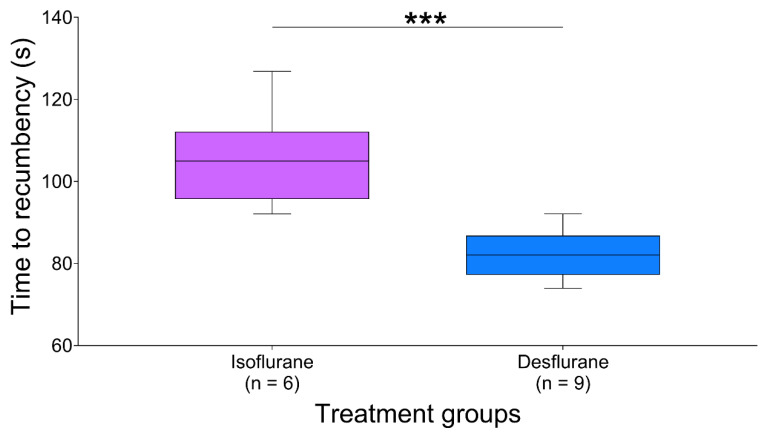
Time to recumbency. During initial exposure, 9/12 and 6/12 rats became recumbent after exposure to desflurane and isoflurane, respectively. Time to recumbency was shorter in rats exposed to desflurane than isoflurane. *** *p* < 0.001. Data are median (middle line in box), IQR (box limits) and 10–90 percentile (whiskers).

Time to recumbency was approximately 20% faster when rats were exposed to desflurane compared to isoflurane during initial exposure (*p* = 0.0008, 95% CI [12.9 to 32.6 s], Figure 2). The number of rats becoming recumbent at initial exposure was 9/12 and 6/12 for desflurane and isoflurane, respectively (*p* = 0.40), with a relative risk of 67% (95%CI 0.3 to 1.3). No rats became recumbent during re-exposure (0/8, both groups). Time from ataxia to recumbency was also shorter during exposure to desflurane (45.0 [34.5–58.0] s), compared with isoflurane (64.4 [45.2–88.4] s, *p* = 0.0048, 95%CI [8.3 to 37.5 s]).

### 3.2. Time to Withdrawal

**Figure 3:**
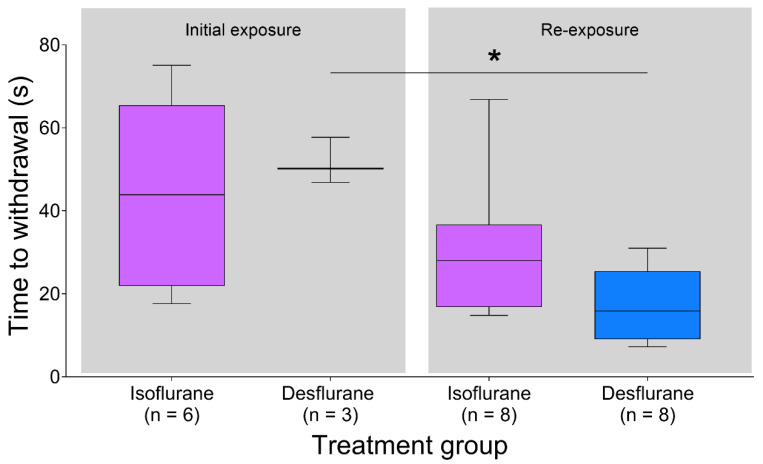
Time to withdrawal was similar for desflurane and isoflurane during both initial and re-exposure. Times to withdrawal between initial and re-exposure was not significant for isoflurane, but was significant for desflurane (*p* < 0.05). Data are presented as median (middle line in box), IQR (box limits) and 10–90 percentile (whiskers). * *p* < 0.05.

During initial exposure, 3/12 and 6/12 rats from the desflurane and isoflurane groups withdrew from the dark chamber, respectively, and this was not different between groups (*p* = 0.40). The time to withdraw from the dark chamber did not differ between desflurane and isoflurane (*p* = 0.714, 95% CI [−24.9 to 34.4 s], Figure 3). During re-exposure, all rats from both groups withdrew (8/8 per group, *p* = 1.00). There was no difference in withdrawal time between anesthetics at re-exposure (*p* = 0.083, 95% CI [−22.2 to 1.1 s]). Withdrawal time was similar during initial and re-exposure of isoflurane (*p* = 0.23, 95% CI [−41.1 to 9.1 s]). Rats exposed to desflurane withdrew sooner at re-exposure compared to initial exposure (*p* = 0.012, 95% CI [20.2 to 46.3 s], Figure 3).

**Figure 4:**
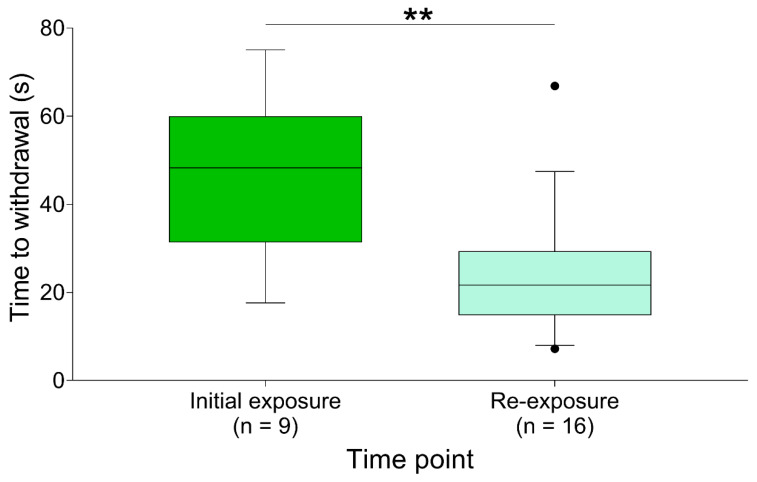
Time to withdrawal pooled by timepoint (initial and re-exposure). Rats withdrew faster upon re-exposure (*p* < 0.01). Sample sizes reflect animals that withdrew. ** *p* < 0.01. Data are presented as median (middle line in box), IQR (box limits) and 10–90 percentile (whiskers). Solid circles indicate outliers (values lying ± 1.5 × IQR).

Pooling the time to withdrawal data by timepoint (initial exposure and re-exposure), revealed that rats withdrew more quickly during re-exposure compared to initial exposure (*p* = 0.004, 95% CI [9.18 to 36.9 s], Figure 4).

### 3.3. Total Dwelling Time

**Figure 5:**
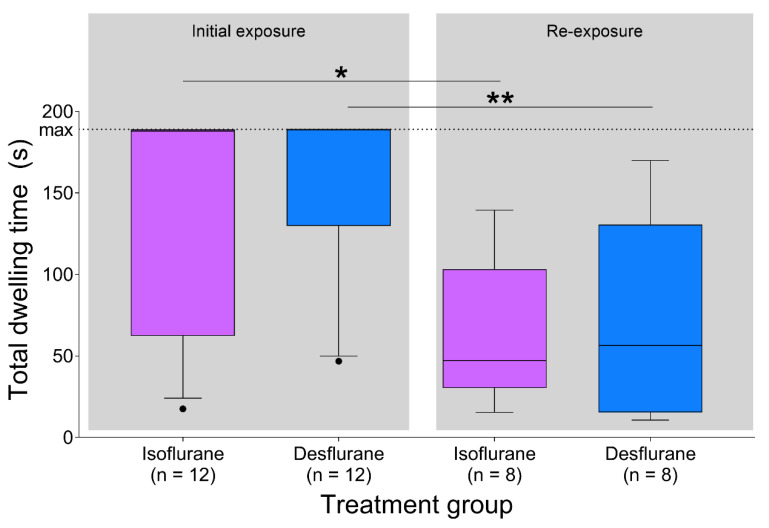
Total dwelling time in dark chamber (site of anesthetic gas exposure) was longer during initial exposure to both desflurane (*p* < 0.01) and isoflurane (*p* < 0.05). During initial exposure and re-exposure, dwelling time was similar between anesthetics (*p* > 0.05). Data are presented as median (middle line in box), IQR (box limits) and 10–90 percentile (whiskers). Horizontal dotted line indicates the maximum duration of testing (3 min). * *p* < 0.05, ** *p* < 0.01. Solid circles indicate outliers (values lying ± 1.5 × IQR).

The total dwelling time in the dark chamber (site of anesthetic gas exposure) was similar for each treatment at both time points: initial exposure (*p* = 0.260, 95% CI [0.0 to 114.0 s]) and re-exposure (*p* = 0.96, 95% CI [−40.6 to 79.9 s], Figure 5). In the desflurane group, total dwelling time was shorter during re-exposure than initial exposure (*p* = 0.002, 95% CI [28.1 to 172.3 s]). The same pattern occurred in the isoflurane group (*p* = 0.022, 95% CI [6.7 to 149.9 s]).

## 4. Discussion

The purpose of this study was to compare the aversiveness of two inhalant anesthetics, isoflurane and desflurane, in laboratory rats. Inhalant anesthetics are commonly administered to induce general anesthesia for procedures, such as surgery, as well as for euthanasia. It is well-established that rats find CO_2_ aversive and potentially painful, leading to calls for refinements and alternative killing methods [3,7,8,9,13,14,15,16,17,18,19,20,21]. Nevertheless, it remains popular and is used more frequently for killing than inhalant anesthetics because it is practical, easy to use, relatively safe for personnel and is a common procedure with established methods [4,14]. In contrast, though in common use for surgical procedures, the delivery of volatile anesthetics requires more equipment, a means to scavenge waste gas and poses a health risk to personnel if used improperly. Where isoflurane is used for euthanasia, it may be as a sole agent (a relatively slow process), or to induce general anesthesia before exposure to CO_2_ [3,4,5].

Previous studies using aversion-avoidance testing have shown isoflurane to be a refinement over CO_2_, as it is less aversive [7]; however, no advantage has been shown with sevoflurane over isoflurane [6]. Using an approach-avoidance test, a lack of difference between sevoflurane and isoflurane has also been reported in mice (male and female C57Bl/6J strain) [22]. In contrast to isoflurane and sevoflurane, which have blood-gas solubility coefficients of 1.4 and 0.7, respectively, desflurane has a coefficient of 0.4 [23]. The blood-gas solubility coefficient determines the speed of onset of inhaled anesthetics, where a lower coefficient reflects a faster increase in partial pressure at the brain, the site of anesthetic action. As expected, exposure to desflurane resulted in a shorter time to recumbency. Though the proportion of animals becoming recumbent at initial exposure was not significantly different between groups, an animal exposed to isoflurane for the first time had a 67% lower chance of becoming recumbent compared to exposure to desflurane. Based on these findings, desflurane appears superior to isoflurane; however, two additional factors should be considered in its use. Firstly, desflurane is approximately ten times more expensive than isoflurane. Additionally, because it is less potent, a higher concentration is required to achieve the same effect. Secondly, desflurane is a greenhouse gas and has a greater global warming potential than either isoflurane or sevoflurane [24].

Furthermore, despite the quicker speed of onset, our data show that desflurane is aversive to rats, to a similar degree as isoflurane. At initial exposure, the rats withdrew from both agents, showing a similar proportion to previous studies with isoflurane and sevoflurane [6,7]. Moreover, learned aversion to both anesthetics was observed when all rats withdrew after re-exposure, seven days after initial exposure. Learned aversion appears to be commonly observed when rats are re-exposed to volatile anesthetics. Previous studies showed this phenomenon when rats were re-exposed 1–2 days after initial exposure [6,7,20]. The presence of aversion after 7 days is relevant as, in the authors’ experience, a 7 days’ interval between procedures requiring general anesthesia is not uncommon. It is unknown if the observed learned aversion would persist with a longer washout period. This is an important consideration when volatile anesthetics are used for killing in instances when animals have previously been exposed to the same, or similar, agents. The stimulus to learning is unknown, but both the smell of volatile anesthetic agents or an unpleasant experience during exposure (including delirium and airway irritation), or both, have been suggested [6,7,20]. Human reports show that both isoflurane and desflurane produce a pungent odor and airway irritation, though isoflurane is better tolerated by humans [10]. In rats, there is evidence that exposure to one inhaled anesthetic may translate to learned aversion for a different inhaled anesthetic, which may reflect an odor common to the inhaled anesthetics [20]. Limiting observations of escape behaviors to a subset of animals was a limitation of the study design. Behavioral observations throughout the study would have made a useful contribution to the dataset and potentially provided further supporting evidence of aversion.

The relationship between chamber volume displacement rates for volatile anesthetics and aversion is relatively unexplored [20,25]. To date, the approach taken has been to increase the concentration relatively quickly, with gas flow rates of approximately 30% of chamber volume per minute used. We speculate that higher flow rates would not only result in a faster induction of anesthesia, but could also prevent the development of learned behavior. However, it is possible that airway irritation is greater during exposure to higher concentrations and exposure to rapidly rising concentrations may be highly aversive [25]. Additionally, the use of high gas flow rates could also be limited by the requirement for waste gas scavenging capable of handling such flows to avoid personnel exposure and the cost associated with increased anesthetic use. In contrast, the use of a lower flow rate would slow induction of general anesthesia, but could elicit a greater degree of aversion and delirium. These possibilities remain to be investigated.

## 5. Conclusions

We have shown that isoflurane and desflurane are similarly aversive; however, desflurane reduced the time to achieve recumbency during initial exposure, shortening any period of potential distress. When re-exposed to either anesthetic, recumbency was never achieved, indicative of aversion.

## Figures and Tables

**Table 1:** Definitions of escape behaviors monitored (after [1]).

**Lid Scratch**	Moving front paw(s) rapidly through 90° angle, from lid downwards.
**Lid Push**	Pushing at the lid using the nose or paw, resulting in lid movement.

**Table 2:** Definitions of time periods.

**Total Dwelling Time**	Total time spent in the dark chamber out of the 3 min trial; includes any time spent in dark chamber after exiting i.e., time spent in the dark chamber following re-entry is included, so that this time may exceed Time to Withdrawal.
**Time to Withdrawal**	Time from start of trial until the initial withdrawal from the dark chamber. Withdrawal defined as both ears passing the plastic strips at the entrance to the dark chamber.
**Time to Recumbency**	Time from start of trial until recumbency, defined as loss of muscle tone, with no purposeful movement and sternal/lateral position with head, tail and all limbs either flat on the bedding or laying against the side of the chamber.
**Time from Ataxia to Recumbency**	Ataxia defined as loss of coordination, including body swaying, unstable foot placement, knuckling, stumbling or falling.
